# Editorial: Stress-induced weight changes

**DOI:** 10.3389/fendo.2023.1209975

**Published:** 2023-05-22

**Authors:** Hassan M. Heshmati, Livio Luzi, Frank L. Greenway, Candida J. Rebello

**Affiliations:** ^1^Endocrinology Metabolism Consulting, LLC, Anthem, AZ, United States; ^2^Department of Endocrinology, Nutrition & Metabolism, MultiMedica Hospital Group, University of Milan, Milan, Italy; ^3^Pennington Biomedical Research Center, Baton Rouge, LA, United States

**Keywords:** stress, stressor, weight change, obesity, weight loss, prevention, treatment

Stress is a universal non-specific response to any pressure or demand. It affects plants, animals, and humans. Stress is a condition caused by a stressor. In humans, the stressor can be physical activity, cold, heat, noise, workload, unemployment, financial difficulties, harassment, racism, domestic violence, separation, divorce, relocation, disease, and deployment to combat. There are differences in the impact of the stressors in relation to genome, gender, age, country, culture, education, social status, and memory. Stress can also be called perceived stress since what is stressful to one person may not be to another. Stress is an experience that is challenging with multiple effects on the physiology and the behavior of humans (1). Stress has been linked to the pathogenesis of numerous medical conditions including depression, hypertension, atherosclerosis, peptic ulcer, osteoporosis, and obesity (2–4). Stress may ultimately lead to death.

Several factors are released during stress (e.g., corticotropin-releasing hormone, arginine vasopressin, adrenocorticotropic hormone, corticosteroids, catecholamines, neuropeptide Y, and inflammatory cytokines). The excess release of these factors may interfere with the normal equilibrium of an individual and cause different disorders. Through complex biochemical changes, stress can induce abnormalities in food intake behavior and fat storage, causing subsequent weight changes (e.g., weight gain or weight loss) (3, 4). The weight gain may lead to obesity. Obesity is a major pandemic responsible for increased morbidity and mortality and high cost for the society (5). The prevalence of obesity is increasing gradually and there is a general perception that life stressors are increasing as well and that the two are related.

The Research Topic on *Stress-Induced Weight Changes* focuses mainly on the relationship between stress and obesity and discusses different underlying mechanisms.

The article published by Eik-Nes et al. reported the relationship between body mass index (BMI) and psychosocial stress, anxiety, and depression in 23,557 adult Norwegian participants (female 55%, mean age 53.8 years, mean BMI 27.2). For the assessment of psychological stress, data were collected on 10 domains such as violence, school bullying, divorce, life-threatening disease, and death. For the measurement of anxiety and depression, the Hospital Anxiety and Depression Scale was used. The study concluded that obesity is significantly related to psychological factors including psychosocial stress and depression.

The Pahk et al. article described the effect of 3 months of physical exercise on amygdala metabolic activity assessed by ^18^F-fluorodeoxyglucose positron emission tomography/computed tomography in 23 women with obesity. Chronic physical exercise reduced stress-associated amygdala metabolic activity and broke its association with systemic inflammation. This research lays the foundation on the mechanism *via* which physical exercise may reduce stress and anxiety. It should be noted that stress-related anxiety and depression are more frequent in women and obesity prevalence is higher in women compared to men. Therefore, this study may suggest a mechanism to explain the gender difference in obesity prevalence. The study would have been more informative if a similar group of men were assessed to look for gender differences in amygdala metabolic activity. The physical exercise program included both aerobic and resistance exercise. It would be interesting to rule out the specific role of aerobic versus anaerobic exercise in amygdala metabolic activity and stress reduction.

The relationship between chronic administration of corticosterone (a stress hormone) and obesity was investigated in the Wang et al. study. The authors found that administration of corticosterone for 8 weeks to 12 mice promotes the proliferation and survival of intestinal cells, possibly contributing to longer small intestines and elongated intestinal villi, leading to increased nutrient absorption and obesity.

The study of Sánchez-Iñigo et al. evaluated BMI fluctuation measured as the average successive variability (ASV) (ASV = |BMIt0 − BMIt1| + |BMIt1 − BMIt2| + |BMIt2 - BMIt3| +…+ |BMItn – 1 − BMItn|/n − 1) and BMI fluctuation rate (ASV/year) in a Caucasian European cohort of 4,312 participants followed for 9.4 years and examined their relationship with cardiovascular events. The study showed that BMI fluctuation rate is associated with increased risk for cardiovascular events and is a better predictor of cardiovascular risk than BMI fluctuation. An unexpected finding was that the association of BMI fluctuation rate with incident cardiovascular disease was greater in people with normal weight or overweight than in those with obesity. While animal studies show that weight cycling reverses the metabolic benefits of weight loss (6), conflicting data from human studies highlight the methodological limitations of the various study designs. Weight loss intervention trials demonstrating long-term benefits may do so due to associated lifestyle changes resulting in improved health outcomes independent of weight. The use of BMI time-rated fluctuation as opposed to BMI or body weight fluctuation is novel and has merit. Nonetheless, the relationship between BMI fluctuation rate and cardiovascular disease risk warrants further investigations.

The administration of an intravenous amino acid is known to stimulate growth hormone (GH) by suppressing somatostatin, and it is used as one of the tests to diagnose GH deficiency. Somatostatin suppression of GH increases as part of the normal aging process, but it is exaggerated during perceived stress. Insulin-like growth factor-1 (IGF-1) is stimulated by the action of GH on the liver, and acts to mediate the actions of GH. A unique oral amino acid mixture was shown to stimulate GH secretion when given in orally administered capsules on an empty stomach (7), and the mechanism of the GH stimulation was demonstrated to be suppression of somatostatin. Fibromyalgia is a known stressor that can cause a reduction in GH and result in stress-related weight gain. The study of Pekarovics et al. demonstrated that the unique oral encapsulated amino acid mixture was able to mitigate stress-related suppression of IGF-1 in 84 participants, give a 6.5% body weight loss over 24 weeks, and improve cardiovascular risk factors with statistical significance without any adverse events.

A better understanding of the stressors and an efficient program aimed to decrease their incidence can help preventing abnormalities in food intake behavior and fat storage, and, therefore, to decrease the occurrence of weight changes, especially obesity and its deleterious consequences.

## Author contributions

All authors listed have made a substantial, direct, and intellectual contribution to the work and approved it for publication.
